# Supplementary material for: Enhancing Acanthamoeba diagnostics: rapid detection of viable Acanthamoeba trophozoites and cysts using viability PCR assay
Source: Microbiol Spectr. 2025 Feb 7;13(3):e01811-24. doi: 10.1128/spectrum.01811-24 (PMC11878052; doi:10.1128/spectrum.01811-24)
Supplement: Table S1 — Ct values and ΔCt values of PMAxx-treated and non-PMAxx-treated samples. [file spectrum.01811-24-s0001.docx]

**Supplementary Table S1.** Ct values and ΔCt values of PMAxx-treated and non-PMAxx-treated samples for both *A. polyphaga* trophozoites and cysts, *A. castellanii* trophozoites, and *A. castellanii* trophozoites from a clinical sample. ΔCt values are calculated by subtracting the Ct value of the PMAxx-treated sample from the non-PMAxx-treated sample. Ct, cycle threshold; ΔCt, delta cycle threshold; *A. polyphaga*, *Acanthamoeba polyphaga; A. castellanii*, *Acanthamoeba castellani.*

|  | *A. polyphaga* trophozoites | | | *A. polyphaga*  cysts | | | *A. castellanii*  trophozoites | | | *A. castellanii* clinical sample trophozoites | | |
| --- | --- | --- | --- | --- | --- | --- | --- | --- | --- | --- | --- | --- |
| % viable  *Acanthamoeba* | Ct value  -PMAxx | Ct value +PMAxx | ΔCt value | Ct value  -PMAxx | Ct value +PMAxx | ΔCt value | Ct value  -PMAxx | Ct value +PMAxx | ΔCt value | Ct value  -PMAxx | Ct value +PMAxx | ΔCt value |
| 100 | 16.95 | 18.04 | -1.09 | 19.76 | 21.48 | -1.72 | 14.22 | 14.37 | -0.15 | 14.99 | 15.41 | -0.42 |
| 50 | 18.49 | 19.25 | -0.77 | 19.26 | 21.12 | -1.86 | 14.70 | 15.18 | -0.48 | 15.72 | 15.94 | -0.22 |
| 10 | 18.67 | 21.42 | -2.75 | 18.74 | 23.15 | -4.41 | 15.29 | 17.54 | -2.24 | 16.91 | 18.31 | -1.40 |
| 1 | 19.16 | 24.85 | -5.69 | 18.69 | 26.75 | -8.05 | 14.83 | 20.44 | -5.61 | 17.25 | 21.08 | -3.83 |
| 0.1 | 18.65 | 27.45 | -8.81 | 18.92 | 28.72 | -9.80 | 15.85 | 24.14 | -8.28 | 17.58 | 22.72 | -5.14 |
| 0 | 18.69 | 31.44 | -12.75 | 18.75 | 29.57 | -10.82 | 15.94 | 28.23 | -12.28 | 18.02 | 28.58 | -10.56 |
